# Does Change in Attention Control Mediate the Impact of tDCS on Attentional Bias for Threat? Limited Evidence from a Double-blind Sham-controlled Experiment in an Unselected Sample

**DOI:** 10.5334/pb.449

**Published:** 2019-01-15

**Authors:** Charlotte Coussement, Pierre Maurage, Joël Billieux, Alexandre Heeren

**Affiliations:** 1 Cellule de Recherche et Publications Scientifiques (CRPS), Hôpital Psychiatrique du Beau Vallon, Namur, BE; 2 Psychological Science Research Institute, Université catholique de Louvain, Louvain-la-Neuve, BE; 3 Clinical Neuroscience Division, Institute of Neuroscience, Université catholique de Louvain, Brussels, BE; 4 Integrative Research Unit on Social and Individual Development (INSIDE), University of Luxembourg, Esch-sur-Alzette, LU

**Keywords:** neuromodulation, transcranial direct current stimulation, attentional bias for threat, attention control, prefrontal cortex

## Abstract

Neurocognitive models of attentional bias for threat posit that attentional bias may result from a decreased activation of the left prefrontal cortex, and especially of its dorsolateral part (dlPFC), resulting in an impaired attention control. Consequently, a transient increase of neural activity within the left dlPFC via non-invasive brain stimulation reduces attentional bias among both anxious and nonanxious participants. Yet, it is still unclear whether the impact of dlPFC activation on attentional bias is mediated by improvement in attention control. In this experiment, we sought to test this hypothesis in an unselected sample (*n* = 20). Accordingly, we adopted a double-blind within-subject protocol in which we delivered a single-session of anodal *versus* sham transcranial Direct Current Stimulation (tDCS) over the left dlPFC during the completion of a task assessing attention control. We also assessed its subsequent impact on attentional bias. Neither attention control nor attentional bias did significantly improve following anodal tDCS. Although our results do not support our main hypothesis, we believe the present null results to be particularly useful for future meta-research in the field. We also formulated a series of methodological recommendations for future research aiming at testing the tDCS-induced modification of attentional bias.

Prominent cognitive theorists of anxiety disorders have argued that attentional bias for threat—that is, a differential attentional allocation for threat-related stimuli relative to neutral ones—figures prominently in the maintenance, and perhaps the etiology, of anxiety disorders (Beck & Clark, 1997; Heimberg, Brozovich, & Rapee, 2010; Mogg & Bradley, 2002; Van Bockstaele et al., 2014; Wong & Rapee, 2016). Accordingly, reducing attentional bias via attention bias modification (ABM) procedures— a computerized training procedure targeting attentional bias—may have clinical benefits (for meta-analyses, see Heeren, Mogoaşe, Philippot, & McNally 2015; Linetzky, Pergamin-Hight, Pine, & Bar-Haim, 2015; Mogoaşe, David, & Koster, 2014). Likewise, transiently increasing attentional bias promotes anxiety proneness among nonanxious individuals (e.g., Heeren, Peschard, & Philippot, 2012; MacLeod, Rutherford, Campbell, Ebsworthy, & Holker, 2002).

The aforementioned studies suggested that attentional bias for threat can be modified and, in turn, reduce anxiety symptoms. Yet, despite these promising initial results, recent meta-analyses indicated that modifying attentional bias via ABM yielded only a very limited impact—albeit significant—on reducing attentional bias and anxiety symptoms (e.g., Cristea, Kok, & Cuijpers, 2015; Heeren, Mogoaşe, et al., 2015; Mogoaşe et al., 2014). However, as pointed out by Grafton & MacLeod (2016), most ABM studies that failed to reduce anxiety also failed to modify attentional bias for threat as intend. Moreover, the mechanisms responsible for ABM remains poorly understood (e.g., Heeren, Coussement, & McNally, 2016; Heeren, De Raedt, Koster, & Philippot, 2013; Mogg & Bradley, 2016). Therefore, the critical next step is thus to improve our understanding of the mechanisms involved in the modification of attentional bias for threat.

One common theoretical explanation for the maintenance of attentional bias focuses on general attention control — i.e. the ability to voluntarily regulate the allocation of attentional resources (Cisler & Koster, 2010; Eysenck & Derakshan, 2011; Heeren, De Raedt, Koster, & Philippot, 2013; Peers et al., 2013). This hypothesis relies on the seminal study of Derryberry and Reed (2002) demonstrating that attention control (assessed using a self-report measure) moderated the magnitude of attentional bias among individuals with elevated trait anxiety—that is, individuals with lower attention control exhibited stronger attentional bias for threat whereas those with higher attention control showed reduced attentional bias. Several replications of this initial study have been reported across distinct paradigms and clinical and nonclinical samples (e.g., Bardeen & Orcutt, 2011; Reinholdt-Dunne, Mogg, & Bradley, 2009; Taylor, Cross, & Amir, 2016). Moreover, changes in attentional bias via ABM procedures depended on the initial level of attention control (Paulewicz, Blaut, & Kłosowska, 2012).

Altogether, these findings dovetail with longstanding neurocognitive models of attentional bias (e.g., Bishop, 2008, 2009; Vuilleumier, 2005) suggesting that the deployment of attention vis-à-vis threatening material is regulated by two primary neural systems: (1) a bottom–up amygdala-based system that produces a signal reflecting the perceived salience of stimuli and directs attention toward salient stimuli (Adolphs, Tranel, Damasio, & Damasio, 1995; Davis & Whalen, 2001), and (2) a top–down system mainly relying on the prefrontal cortex (PFC) that produce a signal when conflicting demands are made on attention and down-regulate amygdala activation in the presence of threat (Bishop, 2004). From this perspective, neuroscientists have hypothesized that attentional bias may result from a failure to recruit regulatory PFC regions that are mandatory to down-regulate amygdala activation in the presence of threat (Bishop, 2009; Britton, Lissek, Grillon, Norcross, & Pine, 2011). Accordingly, reduced left PFC activations, especially of its dorsolateral (dlPFC; Bishop, 2009) and ventrolateral (vlPFC; Monk et al., 2006, 2008) sections, have been found among anxious individuals when completing tasks requiring such a top-down control in the presence of threat. Likewise, PFC areas directly regulate amygdala activity during threat processing (e.g., Monk et al., 2008). Moreover, the reduction of attentional bias via ABM is associated with increased activation of the left dlPFC among healthy volunteers (Browning, Holmes, Murphy, Goodwin, & Harmer, 2010). Likewise, ABM increased vlPFC (Taylor et al., 2014) and attenuated bilateral amygdala activations (Britton et al., 2013; Månsson et al., 2013; Taylor et al., 2014) in patients with anxiety disorders.

Convincing evidence regarding the causal influence of PFC-areas in the maintenance of attentional bias also arise from experimental studies whose manipulation directly targeted the activity of these brain regions during threat processing. Accordingly, a single session of high-frequency repetitive Transcranial Magnetic Stimulation (HF-rTMS) applied over the left dlPFC subsequently decreased attentional bias in a sample of nonanxious healthy participants (De Raedt et al., 2010). Likewise, boosting the activity of the left dlPFC via transcranial Direct Current Stimulation (tDCS)—another noninvasive method of brain stimulation allowing the modulation of the cortical activities *during* the completion of a task—did mitigate attentional bias for threat among patients with a DSM-5 diagnosis of social anxiety disorder (Heeren et al., 2017). Moreover, results indicated that, among healthy undergraduate volunteers, combining tDCS over the left dlPFC with ABM yielded larger reduction in attentional bias than a sham stimulation combined with ABM (Clarke, Browing, Hammond, Notebaert, & MacLeod, 2014; Heeren, Baeken, Vanderhasselt, Philippot, & De Raedt, 2015).

However, despite increasing research linking attentional bias for threat, attentional control, and PFC activations, there are several limitations to the previous studies. First, prominent models of attentional systems postulate that attention control is a multifaceted construct (e.g., Petersen & Posner, 2012; Posner & Rothbart, 2007), including at least three distinct attentional networks: alerting (i.e., maintenance of alertness), orienting (i.e., selective engagement and disengagement with certain stimuli rather than others), and an executive component (i.e., top-down control of attention exemplified by maintenance of attention on certain stimuli and resisting distraction by other stimuli). However, although some research have suggested that the three attentional networks might be distinctively associated with processes assumedly involved in the maintenance of anxiety and related psychopathology (e.g., Heeren, Maurage, & Philippot, 2015; Heeren & McNally, 2016), prior research in the field of attentional bias for threat have almost exclusively treated attention control as a unitary construct and did not differentiate the attentional networks (for a discussion, see Heeren, Billieux, Philippot, & Maurage, 2015). Second, although previous research has suggested that PFC-related areas might be considered as proxy of attention control, there is no study directly testing whether attention control improvement does indeed mediate the impact of PFC-modulation on attentional bias for threat. This is especially unfortunate given the current trends in the development of neurocognitive therapeutic procedures aiming at directly targeting attentional bias to mitigate anxiety (e.g., Clarke et al., 2014; Heeren et al., 2017).

Accordingly, in the present study, we sought to clarify the exact attention control mechanisms whereby the modulation of PFC-areas via tDCS mitigates attentional for threat. This study therefore represents a critical step towards elucidating the mechanism whereby left dlPFC activation via anodal tDCS may mitigate attentional bias. To do so, we adopted a double-blind within-subject protocol in which we delivered single-session of anodal *versus* sham tDCS over the left dlPFC during the completion of a task assessing attention control and, subsequently to the stimulation, on a second task indexing attentional bias for threat. Following the aforementioned HF-rTMS (e.g., De Raedt et al., 2010) and tDCS studies (Heeren et al., 2017), we decided to stimulate the left dlPFC, and not the left vlPFC. Of primary interest was to test whether the reduction of attentional bias for threat following anodal tDCS is mediated by improvement in attention control, as compared to sham tDCS. Moreover, given extant publications of cognitive models bridging executive control to attentional bias for threat (for a review, see Heeren et al., 2013) and the previous observation that the neuromodulation of dlPFC can foster the executive component of attention control (Miler, Meron, Baldwin, & Garner, 2017), we reasoned that if anodal tDCS mitigates attentional bias for threat via improvement of attention control, then this mediational effect should be particularly substantiated for the executive component of attention.

## Method

### Participants

The sample consisted of 20 Caucasian participants (65% females). All participants were right handed. They were recruited via flyers posted in the Université catholique de Louvain community. Exclusion criteria included metal or electronic implants, epilepsy, pregnancy, cardiovascular disease, lifetime history of psychiatric/alcohol/drug dependence, current pharmacological or psychological treatments, corrective eyewear for altered vision, and insufficient knowledge of French language. These criteria were verbally assessed through a medical interview. Participants’ characteristics appear in Table 1.

**Table 1:** Demographic and clinical characteristics of the participants.

	Mean (*SD*)	Cronbach’s alpha

**Demographic measures**		
Age	24.45 (2.70)	
Educational level (in years)	16.95 (2.37)	
**Clinical measures**		
BDI-II	6.20 (7.24)	.92
STAI-T	40.1 (7.32)	.82
LSAS	42.30 (17.20)	.91

***Note:*** Education level was assessed according to the numbers of years of education completed after starting primary school. Ages ranged from 19 to 29 years old. Cronbach’s alphas were computed over the data of the current sample. BDI-II, Beck Depression Inventory; STAI-T, Spielberger State-Trait Anxiety Inventory-Trait version; LSAS, Liebowitz Social Anxiety Scale.

The research was approved by the Ethical Committee of the Medical School of the Université catholique de Louvain (Belgium) and carried out according to the Declaration of Helsinki. Participants provided informed consent, were debriefed upon completion, and were compensated 30€ for their participation.

### Measures

***Descriptive measures.*** To best characterize our sample, participants completed the Beck Depression Inventory (BDI-II; Beck, Steer, & Brown, 1998), the self-report version of the Liebowitz Social Anxiety Scale (LSAS; Liebowitz, 1987), and the Trait Anxiety Inventory (STAIT-T; Spielberger, Gorsuch, Lushene, Vagg, & Jacobs, 1983). Those scales were administered prior to start the experiment. The BDI-II is a 21-item self-reported questionnaire measure of symptoms of depression. The LSAS is a 24-item scale that measures fear and avoidance experienced in a range of social and performance situations over the last two weeks prior to completion. The STAI-T is a 20-item self-report questionnaire assessing anxiety proneness. We used the validated French version of these scales (BDI, Beck et al., 1998; STAI-T, Bruchon-Schweitzer & Paulhan, 1993; LSAS, Heeren, Maurage, et al., 2012). Cronbach’s alphas among the current sample are presented in Table 1.

***Attention networks task (ANT)*.** The efficiency of three independent attentional networks (i.e. alerting, orienting, and executive control) was assessed using the ANT (Fan, McCandliss, Sommer, Raz, & Posner, 2002). Participants had to determine as quickly and accurately as possible the direction of a central arrow (the target) located in the middle of a horizontal line projected either at the top or at the bottom of the screen. They responded by pressing the corresponding button (left or right) on the keyboard. Each target was preceded by either no cue, a center cue (an asterisk replacing the fixation cross), a double cue (two asterisks, one appearing above and one below the fixation cross), or a spatial cue (an asterisk appearing above or below the fixation cross and indicating the location of the upcoming target). Moreover, flankers appeared horizontally on each side of the target. There were three possible flanker types: either two arrows pointing in the same direction as the target (congruent condition), two arrows pointing in the opposite direction of the target (incongruent condition), or two dashes (neutral condition). Each trial had the following structure: (1) a central fixation cross (random duration between 400 and 1600 ms); (2) a cue (100 ms); (3) a central fixation cross (400 ms); (4) a target and its flankers, appearing above or below the fixation cross (the target remained on the screen until the participant responded or for 1700 ms if no response occurred); (5) a central fixation cross [lasting for 3500 ms minus the sum of the first fixation period’s duration and the reaction time (RT)]. RT (in ms) and accuracy (percentage of correct responses) were recorded for each trial.

The ANT task comprised 288 trials, divided in three blocks of 96 trials each (with a short break between blocks). There were 48 possible trials, based on the combination of four cues (no cue, center cue, double cue, spatial cue), three flankers (congruent, incongruent, neutral), two directions of the target arrow (left, right) and two localizations (upper or lower part of the screen). Trials were presented in a random order and each possible trial was presented twice within a block. The task was programmed and presented via E-Prime 2.0 Professional® (Psychology Software Tools, Pittsburgh, PA, USA).

***Probe discrimination task.*** To assess attentional bias, we used a probe discrimination task modeled on the dot-probe detection task (MacLeod, Mathews, & Tata, 1986). The task consisted of 320 trials delivered in one block. Each trial began with a central fixation cross which appeared on the screen for 500 ms. Immediately following the disappearance of the cross, a pair of faces appeared on the screen for 500 ms. One face appeared on the top of center screen, whereas the other face appeared on the bottom of center screen. Each pair of faces displayed neutral-disgust facial expressions.

Immediately after their disappearance, a probe appeared in the location previously occupied by one of the two faces. Participants were asked to indicate whether the probe was a dot (i.e.“.”) or a colon (i.e. “:”) by pressing a corresponding button using the right hand as quickly and accurately as possible. They were also instructed to look at the fixation cross at the start of each trial. The probe remained on screen until a response was given. The inter-trial interval was 1500 ms. There were an equal number of trials for each type of stimuli location (top or down), probe location (top or down), and probe type (“.” or “:”). We used an equal number of trials in each condition as a function of these parameters (i.e., 320 trials = 40 face-pairs × 2 face positions × 2 cue types × 2 cue positions). Each of the 320 trials appeared in a different random order for each participant and each type of stimulation (anodal tDCS versus sham). Stimuli consisted of 40 different face pairs (20 male, 20 female), each pair displaying neutral-disgust facial expressions, randomly selected from a validated version (Goeleven, Raedt, Leyman, & Verschuere, 2008) of the Karolinska Directed Emotional Faces (Lundqvist, Flykt, & Öhman, 1998), which is a standardized set of Caucasian emotional faces. Faces were standardized for size (326 × 329 pixels). The task was programmed and presented using E-Prime 2.0 Professional® (Psychology Software Tools, Pittsburgh, PA, USA).

### Transcranial direct current stimulation

Direct electrical current was delivered by a battery-driven stimulator (Neuroconn, GmbH, Ilmenau, Germany) and applied via a saline-soaked pair of surface sponge rubber electrodes (35 cm^2^). We used a sham-controlled within-subject design in which all participants serve as their own control, a design that substantially increases statistical power. To stimulate the left dlPFC, the anode electrode was vertically positioned centered over the F3 according to the 10–20 international system for electroencephalogram electrode placement. The reference electrode (i.e., the cathode) was placed vertically at the ipsilateral arm (Cogiamanian, Marceglia, Ardolino, Barbieri, & Priori, 2007; Priori et al., 2008). During the first 30 seconds of stimulation, the current was ramped up to 2 mA and then delivered constantly for 25 minutes. At the end of the stimulation, the current was ramped down to 0 mA over 30 seconds. For the sham stimulation, the position of the electrodes was identical to the anodal stimulation; however, the current was ramped down after 30 seconds. This procedure is commonly used in tDCS research and is known to be an optimal way to provide the initial sensation of stimulation without the subsequent effects on cortical excitability (Nitsche et al., 2008; Ohn et al., 2008). Predefined codes assigned to either sham or real stimulation were used to start the stimulator and thus allowed for a double-blind study design. Anodal stimulation, or sham stimulation, respectively, started 5 minutes before the beginning of the ANT and was delivered for a further 20 minutes. Thus, the ANT was performed parallel to the stimulation and the probe discrimination was performed after the stimulation. To be consistent with previous tDCS studies in the field (e.g., Fregni et al., 2005; Heeren et al., 2017), the second stimulation was carried out after an exact 48h-interval to avoid carry-over effect. The order of the anodal and sham stimulation was counterbalanced across participants.

### Procedure

Participants first filled in the questionnaires. Then, the two stimulation-sessions were conducted. At the beginning of each stimulation, electrodes were soaked in saline solution and placed on the participant’s scalp using the electrode montage depicted above. Following 5 min of stimulation (anodal or sham tDCS), participants started with the ANT. The ANT lasted approximately 20 minutes. Immediately after the stimulation, participants started with the probe discrimination task. The probe discrimination task lasted approximately 15 minutes. Participants were asked to perform both tasks as quickly and accurately as possible. The order of the two tDCS-stimulation conditions was randomly counterbalanced across participants (i.e. 10 participants received the anodal stimulation first, 10 participants received the sham stimulation first), and the second stimulation was carried out after an exact 48h-interval. Each session was administrated individually in a dimly lit and quiet room.

## Preprocessing and data analytic plan

### Power analysis

An a priori power analysis was conducted to determine the appropriate total sample size for testing hypotheses with the primary outcome variables. Based upon previous tDCS studies on attentional networks (e.g., Miler et al., 2017) and attentional bias fort threat (e.g., Heeren et al., 2017), we expected a medium-to-large effect size of Cohens’ *d* = 0.7. Setting the level of α at 0.05, power (1 – β) at 0.80 and expecting a conservative correlation of ρ = 0.50 between repeated measurements, the power analysis (G*Power 3.1.3, Faul, Erdfelder, Lang, & Buchner, 2007) indicated that a total sample size of 18 participants would yield an adequate power.

### Data reduction

***ANT.*** Following previous studies (e.g., Heeren et al., 2014; Lannoy et al., 2017; Maurage et al., 2014, 2017), we excluded data from trials with incorrect responses and RTs lower than 200 ms or greater than 2000 ms for each participant at each session. Following Fan *et al*. (2002), we computed the *alerting* effect by subtracting the mean (i.e., RT or accuracy score) for double cue trials from the mean for no cue trials (No cue – Double cue); the *orienting* effect by subtracting the mean for spatial cue trials from the mean result for center cue trials (Center cue – Spatial cue); and the *executive conflict* effect by subtracting the mean for congruent trials (summed across cue types) from the mean for incongruent trials (Incongruent – Congruent). For both alerting and orienting effects, greater subtraction scores for RT indicate greater efficiency. In contrast, greater subtraction scores for RT on executive conflict indicate increased difficulty with executive control of attention (Fan, McCandliss, Fossella, Flombaum, & Posner, 2005).

***Probe discrimination task.*** First, trials with incorrect responses were excluded (2.59% of all the trials following sham; 2.83% of all the trials following anodal tDCS). Second, RTs lower than 200ms or greater than 2000ms were removed from analyses (0.72% of all the trials following sham; 0.70% of all the trials following anodal tDCS). Then, to assess attentional bias, we calculated a bias score for each participant at each session by subtracting the mean latencies when the probe appeared in the same location as the threatening stimuli from the mean latency when the probe and the threatening stimuli appeared at different locations. Hence, positive bias scores represent attention bias toward social threat, and negative bias scores represent bias away from threat, or equivalently, bias toward neutral faces. This bias score is the most frequently used index to determine attentional bias from a probe discrimination task procedure (e.g., MacLeod et al., 1986; Mogg, Philippot, & Bradley, 2004).

### Data analytic plan

To investigate the impact of tDCS on the three ANT networks, we first computed a 2 (Stimulation) × 3 (attention networks) repeated-measures ANOVAs for RT with *Stimulation* (anodal tDCS, Sham) and *Attention Network* (Alerting, Orienting, Executive Conflict) with repeated measurement on the two factors and latencies as dependent variable. To investigate the impact of tDCS on attentional bias, we then computed a paired *t*-test to compare bias scores following sham and anodal stimulation.

Following previous studies in the field (e.g., Fregni et al., 2005), we also examined potential stimulation-order effect. To do so, we computed a 2 (Stimulation: anodal *versus* sham) × 3 (Attentional Networks: Alerting versus Orienting versus Executive control) × 2 (Order: anodal first *versus* sham first) ANOVA with repeated measurement on the first two factors and ANT latencies as dependent variable. Likewise, we computed a 2 (Stimulation: anodal *versus* sham) × 2 (Order: anodal first *versus* sham first) ANOVA with repeated measurement on the first factor and probe discrimination’s *d* scores as dependent variable.

All statistical analyses were performed using SPSS software package (version 20.0). The significance level was set at an alpha level of .05 (bilateral). Effect sizes are reported in the form of partial eta-squared (η^2^_p_) for ANOVA and Cohen’s *d* using the formula for paired comparison (i.e. mean pairs difference divided by the pooled *SD*).

## Results

### Change in attentional networks

The *Stimulation* × *Network* interaction was not significant, *F*(2,38) = 0.07, *p* = .93, η^2^_p_ < .01, implying that the stimulation did not modulate the attentional networks. Likewise, the main effect of *Stimulation, F*(1,19) = .24, *p* = .63, η^2^_p_ = .01, was not significant. Yet, consistent with earlier studies, the main effect of *Network, F*(2,19) = 130.04, *p* < .0001, η^2^_p_ = .87, was significant, implying that the three networks did differ. Results are shown in Table 2.

**Table 2:** Differential latencies (in milliseconds) for bias scores and the three attentional networks as a function of the stimulation.

	Sham stimulation Mean (*SD*)	Anodal tDCS Mean (*SD*)

Alerting	28.42 (18.44)	31.27 (12.43)
Orienting	33.83 (21.79)	34.53 (16.29)
Executive	97.16 (25.67)	97.61 (23.09)
Bias score	–.87 (9.32)	.46 (13.04)

### Change in attentional bias

As shown in Table 2, there was no significant difference regarding attentional bias between anodal and sham stimulations, *t*(19)= .34, *p* = .74, *d* = .33. A comparison between mean latencies when the probe appeared in the same location as the threatening stimuli and the mean latency when the probe and the threatening stimuli appeared at different locations indicated that there was no significant difference between latencies of the former relative to the latter following the sham condition, *t*(19) = 0.46, *p* = .88, *d* = .10 (hence, no attentional bias in the absence of stimulation, i.e. baseline). Similarly, there was no attentional bias for threat following the anodal stimulation, *t*(19) = 0.12, *p* = .90, *d* = .03.

### Stimulation-order effect

The ANOVA revealed a non-significant *Order* × *Stimulation* × *Network* interaction for the ANT, *F*(2,38) = 2.06, *p* = .14, η^2^_p_ = .10. Likewise, the *Order* × *Stimulation* interaction was not significant for the probe discrimination task, *F*(1,19) = .20, *p* = .66, η^2^_p_ = .01. These results confirmed that the present findings did not mirror a stimulation-order effect.

## Discussion

The main aim of the present study was to examine the mediational role of attention control in the impact of anodal tDCS over the left dlPFC on attentional bias for threat, as compared to sham tDCS. Neither attention control nor attentional bias did improve following anodal tDCS. As such, our findings are at odds with previous observation among healthy volunteers of dlPFC-based beneficial impact of non-invasive brain stimulation procedures on attentional bias for threat (e.g., De Raedt et al., 2010). Likewise, our results are also at odds with the previous observation that a 20-minute anodal tDCS over the left dlPFC was associated with greater executive network of the attention in healthy participants (Miler et al., 2017). Altogether, our failures to replicate these findings rendered unstable our main hypothesis that the improvement in attention control mediates the impact of anodal tDCS on attentional bias for threat. There are various potential explanations for our failure to replicate.

First, as pointed out in the results section, our participants did not exhibit an attentional bias in the absence of stimulation—that is, following the sham stimulation. This is unfortunate as several studies indicated that the presence of attentional bias can be considered as a critical factor for the plasticity of attentional bias (e.g., Heeren, Philippot, & Koster, 2015; Kuckertz et al., 2014; Mogoaşe et al., 2014). On the other hand, the observation of a significant change in the magnitude of attentional bias for threat following the combination of anodal tDCS and ABM procedure among participants who were explicitly selected to not possess an attentional bias for threat tends to rule out the hypothesis that the modification of attention bias for threat does mandatorily requires the presence of an attentional bias at baseline (Clarke et al., 2014). Likewise, a recent meta-analysis revealed that, in average, clinical anxious individuals enrolled in randomized controlled trials for ABM are not characterized by attentional bias for threat (Kruijt, Parsons, & Fox, 2018). Although this observation suggests that the beneficial impact of ABM on anxiety symptoms may operate via pathways other than through attentional bias for threat (e.g., Kraft, Jonassen, Heeren, Harmer, Stiles, & Landrø, in press), it also seemingly challenge the claim that anxiety is associated with attentional bias for threat. In our sample, the lack of significant correlation between trait anxiety scores and attentional bias for threat in the absence of stimulation, *r*(20) = .15, *p* = .52, dovetails with this claim. Yet, most of those studies were conducted among individuals with clinical anxiety disorders and trait anxiety score might be less than ideal to depict anxiety and related psychopathology (e.g., Heeren, Bernstein, & McNally, 2018). As such, it remains particularly difficult to interpret the absence of attentional bias in the present sample without an anxious comparison group.

Second, we used a dot-probe task to assess attentional bias for threat. Yet, like most extant procedures for assessing attentional bias, the dot-probe task exhibits poor psychometric properties (for a recent review, see McNally, 2018). Recently developed experimental paradigms enabling optimal assessment of attentional bias might be more appropriate in future research agendas (e.g., Price et al., 2015; Sanchez-Lopez, Vanderhasselt, Allaert, Baeken, & De Raedt, 2018; Zvielli, Bernstein, & Koster, 2014).

Third, we chose disgust faces as threat cues. The rationale behind our decision was twofold. First, disgust conveys a message of aversion or rejection in both clinical and nonclinical samples (Rozin, Lowery, & Ebert, 1994). Second, previous studies supporting the effectiveness of either anodal tDCS or ABM in reducing attentional bias for threat relied on faces expressing disgust as threatening stimuli (e.g., Heeren et al., 2017; Pieters et al., 2016; Sanchez-Lopez et al., 2018). However, other studies have relied on fearful or angry faces as threat cues for assessing attentional bias for threat via the dot-probe task (for a review, see van Rooijen, Ploeger, & Kret, 2017). As such, one cannot exclude that the findings would have been radically different using fearful or angry faces as threat cues. Accordingly, future research could examine whether the impact of anodal tDCS on attentional bias for threat does vary across different types of threat cues.

Fourth, we used the ANT to assess attention control. Yet, most of the attentional bias research has assessed attention control using the Attention Control Scale (Derryberry & Reed, 2002), a self-report measure assessing attention control as a trait-like construct. As such, one cannot exclude that our findings would be been different using the Attention Control Scale. On the other hand, the examination of the impact of a single session of tDCS on a self-report measure has no relevance, especially given the trait-like nature of attention control as assessed using the Attention Control Scale. Moreover, the ANT has been repeatedly used in anxiety research (e.g., Heeren, Maurage, & Philippot, 2015; Moriya & Tanno, 2009; Pacheco-Unguetti et al., 2011) and has been already used in tDCS research (e.g., Miler et al., 2017). Likewise, prior research has shown that the distinct ANT’s attentional networks, and particularly the executive conflict index, were strongly associated with attentional bias for threat (e.g., Enock, Hofmann, & McNally, 2014; Heeren & McNally, 2016; Heeren, Mogoaşe, McNally, Schmitz, & Philippot, 2015). The presence of a significant correlation between the executive conflict index and attentional bias for threat in the absence of stimulation, *r*(20) = .46, *p* < .05, corroborated this observation in our sample.

Fifth, while previous tDCS studies administered the probe discrimination task during the stimulation, ours did after. Although we decided to set up our experiment in accordance to our mediational hypothesis vis-à-vis the potential impact of dlPFC-induced attention control improvement on attentional bias mitigation, to the best of our knowledge, no previous studies did collect post-stimulation data. Accordingly, our failure to replicate the tDCS-induced mitigation of attentional bias may merely mirror the non-persistence of the dlPFC-induced benefits at post-stimulation, that is, during the completion of the probe discrimination task. Consequently, an important next step would thus be to compare the *online* versus *offline* tDCS-induced mitigation of attentional bias for threat in anxious and nonanxious samples.

Sixth, although our decision to target the left dlPFC relied on previous rTMS and tDCS studies (Clarke et al., 2014; De Raedt et al., 2010; Heeren, Baeken, et al., 2015; Heeren et al., 2017), vlPFC has been also associated with attentional bias for threat and anxiety (Fox & Pine, 2012; Hartley & Phelps, 2010; Heeren, Dricot, et al., 2017). Consequently, one cannot exclude that vlPFC would have been a better target. On the other hand, several reviews and meta-analysis suggested that left dlPFC does constitute an optimal target to improve top-down control processes among healthy volunteers (e.g., Brunoni & Vanderhasselt, 2014). Future studies should thus further delineate the respective contribution of both ventral and dorsal compartments of PFC vis-à-vis the modification of attentional bias in clinical and nonclinical samples. In the same vein, although our decision to not rely on bipolar-balanced montage—that is, the anode centered over the left dlPFC and the cathode centered over the right dlPFC—was based upon prior research in the field of attentional bias (e.g., Clarke et al., 2014; Heeren, Baeken, et al., 2015; Heeren et al., 2017) and ANT (e.g., Miler et al., 2017), different montage may have yielded various outputs. Especially, the position of the reference electrode—in the present case, the cathode— may have impacted on the overall current flow pattern as the wider the distance between the two electrodes, the smaller the current density under the electrodes (e.g., DaSilva, Volz, Bikson, & Fregni, 2011; Moliadze, Antal, & Paulus, 2010). In this way, although we relied on an extra-cephalic placement for the reference electrode to avoid any cortical influence of the cathode, this montage may have reduced the overall current density. Future experiments are thus also needed to clarify this issue.

Finally, our *a priori* power analysis was based upon previous tDCS studies on attentional networks (e.g., Miler et al., 2017) and attentional bias (e.g., Heeren et al., 2017). However, although our sample size had adequate power to detect medium effect sizes, our analysis would have benefited from a larger sample size. On the other hand, neither the *p-*values nor the effect sizes associated with our nonsignificant effects even approaches statistical significance. Moreover, a complementary power analyses indicated that a total sample size of at least 787 participants would be required to yield enough power to detect a small effect size (i.e., Cohen’s *d* = .10) in the present study. However, such small effect sizes have extremely limited relevance for translational research.

In conclusion, these limitations notwithstanding, this study constitutes, to the best of our knowledge, the first attempt to test whether attention control mediates the impact of dlPFC-based anodal tDCS on attentional bias for threat. Although our findings do not dovetail with prior research, we proposed several potential explanations for our failure to replicate. Altogether, we believe the present null findings study to be particularly useful for future empirical investigation and meta-research in the field.
